# Genome-Wide Mutation Rate Response to pH Change in the Coral Reef Pathogen *Vibrio shilonii* AK1

**DOI:** 10.1128/mBio.01021-17

**Published:** 2017-08-22

**Authors:** Chloe Strauss, Hongan Long, Caitlyn E. Patterson, Ronald Te, Michael Lynch

**Affiliations:** Department of Biology, Indiana University, Bloomington, Indiana, USA; University of Texas at Austin

**Keywords:** environmental dependence of mutations, evolutionary genomics, mutation accumulation, neutral evolution

## Abstract

Recent application of mutation accumulation techniques combined with whole-genome sequencing (MA/WGS) has greatly promoted studies of spontaneous mutation. However, such explorations have rarely been conducted on marine organisms, and it is unclear how marine habitats have influenced genome stability. This report resolves the mutation rate and spectrum of the coral reef pathogen *Vibrio shilonii*, which causes coral bleaching and endangers the biodiversity maintained by coral reefs. We found that its mutation rate and spectrum are highly similar to those of other studied bacteria from various habitats, despite the saline environment. The mutational properties of this marine bacterium are thus controlled by other general evolutionary forces such as natural selection and genetic drift. We also found that as pH drops, the mutation rate decreases and the mutation spectrum is biased in the direction of generating G/C nucleotides. This implies that evolutionary features of this organism and perhaps other marine microbes might be altered by the increasingly acidic ocean water caused by excess CO_2_ emission. Nonetheless, further exploration is needed as the pH range tested in this study was rather narrow and many other possible mutation determinants, such as carbonate increase, are associated with ocean acidification.

## IMPORTANCE

This study explored the pH dependence of a bacterial genome-wide mutation rate. We discovered that the genome-wide rates of appearance of most mutation types decrease linearly and that the mutation spectrum is biased in generating more G/C nucleotides with pH drop in the coral reef pathogen *V. shilonii*.

## INTRODUCTION

One of the main avenues of species diversity is mutational change. Despite the high species diversity and abundance of marine microbes (reviewed in reference 1), the association between their spontaneous mutations and environmental factors is rarely studied. This sharply contrasts with recent research progress in the area on bacteria from other habitats (2, 3). The mutation accumulation (MA) technique is currently one of the most accurate methods for studying genome-wide spontaneous mutations, applying repeated single-individual bottlenecks to large numbers of parallel lineages over hundreds or even thousands of generations (4, 5). The efficiency of selection is greatly weakened by strong population bottlenecks during this process, whereas genetic drift is promoted and dominates selection. Thus, all mutations can be accumulated in a virtually neutral fashion except those with extremely large fitness effects. Deep whole-genome sequencing combined with bioinformatic tools further facilitates the use of this experimental procedure in detecting genome-wide mutations in mutation accumulation (MA) lines directly and accurately (6).

Ocean acidification is one major challenge of global climate change. Decreases in the pH of seawater during this process can affect bacterial growth and evolution. The genome-wide mutational responses of pathogenic microbes to pH, a potentially key parameter in host-parasite coevolution in aquatic systems, are essentially unstudied *in vivo*. *In vitro* studies have indicated that low pH increases the efficiency of error removal by the exonuclease-deficient form of the Klenow fragment of *Escherichia coli* DNA polymerase I: from pH 9.8 to 6.2, frameshift (insertion or deletion) and base-substitution mutation rates are decreased by 40-fold and 50-fold, respectively, possibly by altering the template-binding properties of the DNA polymerase (7).

Members of the genus *Vibrio* are common pathogens in marine environments. The bacterium *Vibrio shilonii* (genome size, ∼5.7 Mbp; the first known *Vibrio* species to attack zooxanthellae) has been found to be a cause of bleaching in the coral *Oculina patagonica* (8–10). It was speculated that the bleaching is stimulated by global warming (9, 11). The bacteria bleach corals at an elevated temperature by adherence of their cells to *β*-d-galactopyranosides on the coral’s surface (11) and then penetration of the tissue and multiplication in number (12). This harms the symbiotic zooxanthellae of the coral by producing toxins that inhibit photosynthesis and lyse the algae (13).

In this project, we performed mutation accumulation experiments on *V. shilonii* AK1 cultured on marine agar plates with various pH values. The goal of this study was to decipher its genome-wide mutation rate and spectrum and its response to pH change. These results may yield clues relevant to the long-term evolution of this key marine pathogen.

## RESULTS

In order to resolve the mutation rate and spectrum of *V. shilonii* at different pH levels, we initiated studies in which single colonies of 84 MA lines were transferred every other day at each pH. The mutation accumulation transfers took about 4 months, equivalent to 1,003 to 1,568 cell divisions (Table 1). For each pH treatment, an average of 53 MA lines with a mean depth of sequencing coverage of ∼74 were used in the final mutation analyses. The control group had an average pH of 8.14 to simulate the pH of natural seawater and to serve as a comparison with the other three treatment groups (pH 7.76, 7.29, and 6.67; *V. shilonii* does not grow at pH levels of <6.5 in our culturing system; Table 1).

**TABLE 1:** Mutation accumulation line details^a^

pH (95% CI)	*N*	Transfers	X	Divisions (G)	Ts	Tv	Ins	Del
8.14 (8.11, 8.17)	52	59	63	1,557 (1.82)	50	46	5	7
7.76 (7.71, 7.81)	53	60	81	1,568 (1.84)	38	27	9	10
7.29 (7.23, 7.36)	52	46	78	1,153 (1.92)	32	19	2	12
6.67 (6.61, 6.73)	54	44	76	1,003 (2.11)	10	8	5	1

a CI, confidence interval from *t* distribution; *N*, number of MA lines used for mutation analysis, after removing lines with low coverage (<15×) or cross-line contamination; Transfers, average number of transfers for each MA line (∼48 h between two consecutive transfers); X, mean depth of coverage of genome sequencing; Divisions (G), mean number of cell divisions for each MA line passed during the experimental span (G, generation time in hours); Ts, total number of transitions pooled from all MA lines in the group; Tv, total number of transversions; Ins, total number of insertions in the group; Del, total number of deletions in the group.

The ratio of nonsynonymous to synonymous base substitutions helps reveal whether mutations are biased by selection. None of the nonsynonymous to synonymous base-substitution ratios in the various experimental groups is significantly different from the random expectation (χ^2^ test, *df* = 1, *P* > 0.05). Thus, the accumulated mutations reflect the true mutagenesis pattern at different pH levels, which is in agreement with analyses of virtually all prior MA experiments involving microbes.

### Genome-wide mutation rate and spectrum of *V. shilonii* at natural seawater pH.

**FIG 1:**
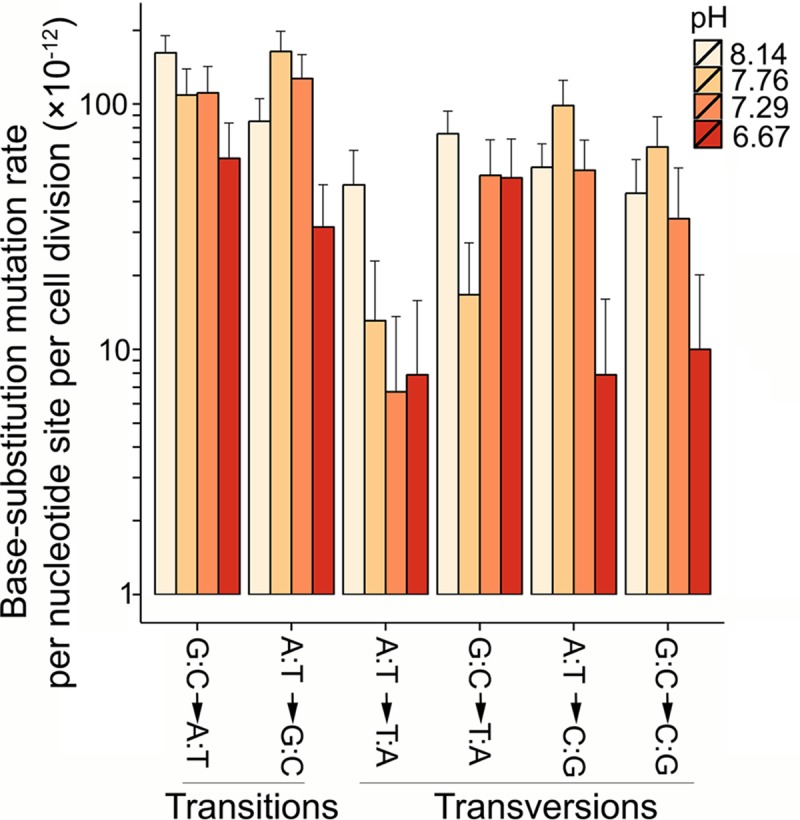
Mutation spectra of *V. shilonii* at different pH levels. Error bars represent standard errors of the mean.

At the control pH of 8.14, 96 base-substitution mutations were detected and led to a base-substitution mutation rate estimate of 2.29 × 10^−10^ ± 0.25 × 10^−10^ (standard error of the mean [SEM]) per nucleotide site per cell division, or 1.18 × 10^−3^ per genome per cell division. This genomic mutation rate is highly similar to those of other *Vibrio* species and many other bacteria from various habitats that have been studied with the MA method (14; reviewed by Lynch et al. [2]). Among the base substitutions, there are 50 transitions and 46 transversions, leading to a transition/transversion ratio of 1.09 (Fig. 1) (Table 1; see also Tables S1 and S2 in the supplemental material). Twelve small-indel mutations (5 insertions, 7 deletions; Tables S1 and S3) were detected, yielding an indel mutation rate of 2.86 × 10^−11^ ± 0.82 × 10^−11^ per nucleotide site per cell division.

10.1128/mBio.01021-17.1 TABLE S1  Mutation statistics of MA lines at different pH levels. Download TABLE S1, XLSX file, 0.1 MB. This content is distributed under the terms of the Creative Commons Attribution 4.0 International license.

10.1128/mBio.01021-17.2 TABLE S2  Base substitutions and functional context. Download TABLE S2, XLSX file, 0.04 MB. This content is distributed under the terms of the Creative Commons Attribution 4.0 International license.

10.1128/mBio.01021-17.3 TABLE S3  Indel details. Download TABLE S3, XLSX file, 0.01 MB. This content is distributed under the terms of the Creative Commons Attribution 4.0 International license.

Similarly to prior results for most A/T-rich bacteria (*V. shilonii* genome A/T content, ∼56%), mutations were biased in the A/T direction in *V. shilonii*, with a mutation rate in the A/T direction (including G·C→A:T transitions and G:C→T:A transversions) of 2.38 × 10^−10^ and in the G/C direction of 1.40 × 10^−10^ (A·T→G:C transitions and A·T→C:G transversions; Fig. 1; Table S1). Given these mutation rates, we estimate that the A/T content of *V. shilonii* under the condition of mutation pressure alone would be 63% (standard error [SE], 15%), which is not significantly different from the actual A/T content of this organism.

### Mutational response to pH decrease.

**FIG 2:**
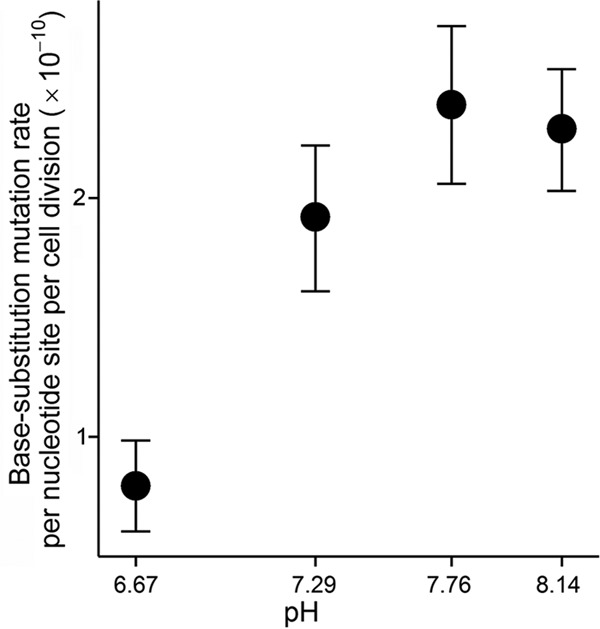
Mutation rate at different pH levels. Error bars represent standard errors of the mean.

A one-sided Pearson’s correlation test shows a strong positive correlation between pH and mutation rate (*r* = 0.92, *P* = 0.04; Fig. 2), with the mutation rate at the highest pH being elevated ∼3× in comparison to that seen at the lowest pH. Compared with the high (∼50×) mutation rate elevation at pH 9.8 versus 6.2 in the *in vitro* study of *E. coli* (7), the limited elevation in living *V. shilonii* cells could be a consequence of possibly more-efficient DNA repair systems at higher pH. We also fitted a generalized linear mixed-effects model to the mutation data set in Table S1, with mutation rate as the response variable, pH as the fixed effect, and MA lines as the random effect nested within each pH group. As shown by the model, the response of mutation rate to pH was about 1.06 × 10^−10^ per pH unit (*P* < 0.0001). The mutation rate variance among the MA lines was also changed by 1.26 × 10^−20^ per pH unit. The mutation rate decrease at low pH was not limited to certain base substitutions but was distributed over most possible base-substitution types (Fig. 1; no conclusion was drawn on indel mutation rate versus pH due to the lack of statistical power; Table S1).

To determine whether pH also alters the mutation spectrum, we categorized the pH 8.14 group as “natural pH” and, in order to increase the statistical power at low pH treatments, we combined the other three pH groups and categorized the combination as the “lower pH” group. Then, we investigated the mutation bias with respect to the A/T direction using the variable *m*, which is calculated as the ratio of the mutation rate in the A/T direction to that in the G/C direction. *m* significantly changed from 1.70 (SE = 0.39) at “natural pH” to 0.79 (SE = 0.15) within the “lower pH” group. Thus, lower pH alters the mutation spectrum of *V. shilonii* in the direction of generating more G/C nucleotides.

The trend of mutation rate decline with lower pH is highly consistent with previous studies reporting that lower pH increases the DNA polymerase replication fidelity of *Escherichia coli* (7) and decreases the nucleotide depurination of *Bacillus subtilis* (15). The mutation spectrum bias to the generation of more G/C nucleotides at lower pH also corresponds well with results of another study, which found that pH decreases caused a lower level of cytosine deamination, the major cause of mutations generating A/T nucleotides (Fig. 1) (16).

## DISCUSSION

The similarity of the genomic mutation rate and spectrum of *V. shilonii* to those of other bacteria indicates that the marine environment, though both chemically and physically different from other bacterial habitats, is not a major determinant of mutation rate evolution. According to the drift-barrier hypothesis (2, 17), effective population size (the metric reflecting the power of random genetic drift) of an organism is the major determinant of evolved mutation rates as it sets the balancing point where the selective advantage of any drop in the mutation rate is insufficient to overcome the power of genetic drift. Future population-genetic studies on natural *V. shilonii* populations are required to test this hypothesis.

Caldeira and Wickett (18) recently predicted that the surface pH of the ocean will decrease to ∼6.5 by the year 2500 given the current rate of CO_2_ emission. This implies that the evolution of *V. shilonii*, and perhaps of other marine species, could be altered by the acidification of seawater over the next 5 centuries, assuming that the evolution would be strongly limited by mutational input, which need not be the case. However, ocean habitats of *V. shilonii* are influenced by numerous additional physical and chemical factors which were not taken into account in this study. Ocean acidification is a complicated process (19), and the introduction of other entities into the water, such as carbonates, might also influence the mutational process. Future exploration of mutational responses to ocean environmental change needs to include such factors.

In conclusion, we deciphered the mutation rate and spectrum of the coral reef pathogen *V. shilonii* at the whole-genome level and found a trend of mutation rate decrease and mutation-spectrum bias in generation of more G/C nucleotides as pH drops. Although numerous efforts were made to keep a stable and accurate pH in each treatment, there is still the possibility that pH might fluctuate during the mutation accumulation process. The maintenance of the pH value inside the *V. shilonii* colonies might be complicated by the presence of secreted metabolites (pH buffers were not used in the current culturing system because of the concern that an uncontrolled buffer salt concentration might influence the mutation process). More experiments using a larger pH gradient and conducted over longer periods of mutation accumulation would provide higher statistical power for studying marine microbial mutations besides base substitutions, such as structural variants.

## MATERIALS AND METHODS

### Strain, media, and transfer.

*V. shilonii* (BAA-91 [the ATCC version of strain AK-1]) was ordered from ATCC. A total of 84 MA lines were established for each of the four pH treatments (8.14, 7.76, 7.29, and 6.67). A total of 83, 79, 68, and 57 MA lines survived to the end of the transfers for pH 8.14, 7.76, 7.29, and 6.67, respectively. Each MA line grew on marine agar plates at 30°C, using the following laboratory-developed recipe: for 1 liter of medium, 25 g Instant-Ocean sea salt, 5 g Bacto peptone (BD), 1 g Bacto yeast extract (BD), 15 g agar (Mooragar bulk agar), and 0.18 g ferric EDTA (ferric sodium salt trihydrate; Acros Organics) (supplemented with deionized water to 1 liter).

The pH of agar plates changes slightly after autoclaving, especially with large-volume containers (for example, 4-liter to 6-liter flasks), and the low pH gradient (0.5) between different treatments could be affected by such changes. In order to accurately control the pH of the marine agar plates, a few procedures were implemented. Either 0.5 M HCl or 0.5 M NaOH was added to 1 liter of liquid medium in 2-liter flasks before agar powder was added, and the pH of each treatment (before autoclaving) was started at 6.30 (for 6.67 treatment), 7.00 (7.29), 7.70 (7.76), and 8.50 (8.14). For each batch of medium preparation, the surface pH of at least three agar plates was measured using a Ross flat-bottom pH electrode, and only plates with a final pH within range (maximum difference, 0.2) were stored at 4°C and used for transfers within 2 weeks. The pH statistics pooled from all measurements are shown in Table 1.

Single-colony transfers were performed every 48 h. The whole MA experiment lasted about 4 months, and each line was transferred approximately 59 (pH 8.14), 60 (7.76), 46 (7.29), or 44 times (6.67) on average. The number of cell divisions between two transfers was estimated approximately every month using CFU counts of 10 randomly selected lines from each treatment. The number of cell divisions between two transfers (*n*) was then calculated as *n* = log_2_ CFU. The total number of cell divisions passed for each line equals the grand mean of all *n* estimates multiplied by the line’s total number of transfers.

### DNA extraction, library construction, and genome sequencing.

DNA was extracted from MA lines from each pH treatment using a Wizard genomic DNA purification kit (Promega, Madison, WI), and DNA libraries were constructed for genome sequencing using a Nextera DNA library preparation kit (Illumina). Size selection was performed for an insertion size of 300 bp, and the lines were sequenced using a HiSeq 2500 system (2 × 150 rapid run at the Hubbard Center for Genome Studies, University of New Hampshire). Totals of 54, 60, 54, and 56 MA lines were sequenced at pH 8.14, 7.76, 7.29, and 6.67, respectively, and 52, 53, 52, and 54 MA lines were eventually used in the final mutation analysis after removal of low-coverage (<15×) or cross-contaminated lines—cross-contamination was identified by lines sharing mutations and being cultured on the same plate or on adjacent plates—(Table 1). The mean depths of coverage were 63× (pH 8.14), 81× (7.76), 78× (7.29), and 76× (6.67)—the details concerning the coverage of each MA line are given in Table S1 in the supplemental material. All raw sequence reads were deposited in NCBI SRA (study no. SRP098907).

### Mutation analyses.

Adaptors in raw reads were trimmed off using Trimmomatic 0.32 (20). Reads were then mapped to the reference genome using BWA-0.7.10 mem (GenBank genome accession number GCF_000181535.1 [ASM18153v1]) (21). Removal of duplicate reads was conducted using Picard-tools-1.141, and realignment of reads around indels was conducted with GATK-3.5. Single nucleotide polymorphism (SNP) and indel discovery was performed with standard hard filtering parameters according to the GATK Best Practices recommendations (Phred-scaled quality score [QUAL] of >100 and root mean square [RMS] mapping quality [MQ] of >59 for both variant and invariant sites) (22–24). Base pair substitutions and small indels were called using UnifiedGenotyper in GATK. Greater than 99% of reads in a line were required to determine the line-specific consensus nucleotide at a candidate site—a level of 1% was set to allow for aberrant reads originating from sequencing errors, impure indices during library construction, or barcode degeneracy during sequence demultiplexing.

The mutation rate µ was calculated as $\text{μ}=\frac{m}{\sum_{i=1}^{n}N_{i}\;\times \;T_{i}}$, where *m* is the total number of mutations pooled from all MA lines of a treatment, *n* is the total number of lines, *N* is the number of analyzed sites in one line, and *T* is the total number of cell divisions that the MA line passed.

Calculation of equilibrium A/T content followed Lynch (25) and was performed using$\frac{u}{u+v}$, where *u* is the mutation rate in the A/T direction (including both G:C→A:T transitions and G:C→T:A transversions) and *v* is the mutation rate in the G/C direction (including both A:T→G:C transitions and A:T→C:G transversions). Mutation bias (*m*) in the A/T direction was calculated using$\frac{u}{v}$. Calculation of the standard error of the equilibrium A/T content and *m* followed the methods of Lynch and Walsh (26).

We used R package nlme for the generalized linear mixed-effects model fitting (lme function) (27).
